# Digital Gaze and Vicarious Trauma Among Intensive Care Unit Nurses in Alarm-Monitoring Ecologies: Qualitative Interview Study

**DOI:** 10.2196/91807

**Published:** 2026-08-04

**Authors:** Yuanyuan Wang, Hongyu Chen, Kui Fang, Xu Guo, Yueqin Gu

**Affiliations:** 1 Department of Intensive Care Unit, The First Affiliated Hospital of Zhejiang Chinese Medical University (Zhejiang Provincial Hospital of Chinese Medicine), Postal Road No. 54, Shangcheng District, Hangzhou, Zhejiang, 310000, China, 86 13958128553; 2 School of Nursing, Chinese Academy of Medical Sciences and Peking Union Medical College, Beijing, China; 3 Department of Neurosurgery, The First Affiliated Hospital of China Medical University, Shenyang, Liaoning, China

**Keywords:** intensive care unit, nurses, clinical alarms, vicarious trauma, patient monitoring, qualitative research

## Abstract

**Background:**

Intensive care units (ICUs) rely on continuous physiological monitoring and frequent alarms to detect patient deterioration. Although alarm fatigue has been widely discussed as a patient safety and workflow issue, less is known about how monitoring systems shape nurses’ attention, visibility, perceived accountability, emotional strain, and recovery after distressing events. Understanding these experiences is important for designing safer monitoring displays, alarm behavior, communication routines, and future AI-supported systems.

**Objective:**

This study aimed to explore how ICU nurses experience continuous monitoring and alarms as a digital work environment, with particular attention to digital gaze, vicarious trauma–related emotional strain, perceived accountability, and design-relevant system needs.

**Methods:**

We conducted a qualitative interview study with 15 ICU nurses from a tertiary hospital in Hangzhou, China. Semistructured interviews probed 6 topics: everyday monitoring routines and alarm exposure; responses to alarms, patient deterioration, and death; perceived pressure related to visible physiological data; after-shift experiences following distressing events; coping and recovery strategies; and suggestions for alarm governance and monitoring system design. Data were analyzed using reflexive thematic analysis.

**Results:**

Four themes were generated. First, continuous monitoring created a form of digital gaze in which nurses maintained constant watch, experienced alarm-driven interruptions, and felt that visible physiological data made bedside responses open to scrutiny. Second, patient deterioration and death were experienced partly through monitoring technologies, including weakening waveforms, escalating alarms, numerical decline, and eventual silence. Third, monitoring-related stress extended beyond the ICU through lingering alarm sounds, monitor images, personal resonance, and work-related messages after shifts. Fourth, participants described recovery strategies but also emphasized system-level needs, including clearer alarm prioritization, fewer nonactionable alerts, gentler auditory design, more useful trend displays, postresuscitation buffering, and better digital communication boundaries.

**Conclusions:**

Continuous monitoring and frequent alarms shaped ICU nurses’ work beyond workflow disruption and patient safety. Alarm-intensive care should be understood as a digital health and sociotechnical design issue that affects attention, perceived accountability, emotional strain, and recovery. Future monitoring systems should be co-designed and evaluated not only for alarm reduction and technical accuracy but also for how displays, alarm behavior, work-related communication, and AI-supported tools affect clinicians’ work, recovery, and perceived surveillance.

## Introduction

Intensive care units (ICUs) are highly digitalized clinical environments in which high-acuity care is organized around continuous physiological monitoring and complex sociotechnical work systems [1,2]. Bedside monitors, ventilators, infusion pumps, and other connected devices generate streams of physiological data, waveforms, device alerts, and audible or visual alarms that nurses must interpret, prioritize, and act on in real time [2,3]. These technologies are central to patient safety because they support the early detection of deterioration but also create a demanding digital work environment in which nurses’ attention is repeatedly redirected by screens, sounds, and alerts. ICUs are alarm-dense settings, and alarm rates vary by patient and clinical characteristics, while many alarms are clinically nonactionable [1,4]. Frequent nonactionable alarms can disrupt nursing work, increase cognitive burden, and reduce confidence in alarm systems [2,3].

Alarm-related risks cannot be addressed through individual vigilance alone. Existing guidance emphasizes alarm policy, parameter configuration, staff education, workflow redesign, and unit-specific implementation [5-7]. Research on ICU nurses’ cognitive ergonomics shows that alarm management is embedded in everyday work, requiring nurses to recognize, interpret, prioritize, reset, or silence alarms while managing competing tasks [8]. Scholarship on the digital nursing gaze and nursing surveillance likewise conceptualizes monitoring as more than measurement: it organizes interpretation, anticipation, continuous visibility, and responsibility-bearing work [9-11]. By making certain physiological changes visible, audible, and urgent, monitoring interfaces and alarm signals shape how nurses allocate attention, interpret patient risk, and coordinate responses [12,13]. They can also become shared reference points for interpreting patient status and organizing action [14]. Continuously visible parameters may heighten nurses’ perceived accountability when deterioration is difficult to reverse [9-11]. These features are therefore not merely operational; they are health system design choices with consequences for both patient safety and nurses’ work.

Within this alarm-intensive and digitally mediated work environment, ICU nurses are repeatedly exposed to patient suffering, rapid deterioration, death, family distress, and emotionally charged clinical encounters. Such experiences have been discussed in relation to compassion fatigue, secondary traumatic stress, and other forms of occupational distress [15,16]. Vicarious trauma is a useful interpretive lens for this study because it concerns enduring changes in helpers’ assumptions about safety, trust, control, and meaning that may arise through empathic engagement with others’ trauma [17,18]. In nursing, vicarious trauma–related effects may arise not only through direct exposure to patient injury and death but also through repeated encounters with family grief, interpersonal conflict, and threats or hostility toward staff [19,20]. In this study, we use vicarious trauma as an interpretive lens for understanding the distress and psychological residue that may accumulate through repeated witnessing of suffering, deterioration, or death during care. Rather than treating monitoring technologies as independent causes of vicarious trauma, this framing directs attention to how they may shape the conditions under which distressing clinical events are witnessed, anticipated, and remembered.

Despite growing attention to alarm fatigue and alarm governance in intensive care [5-7], alarm fatigue research has largely focused on alarm burden, response behavior, nuisance reduction, and safety outcomes. Less is known about how ICU nurses experience continuous monitoring and alarms as a digital work environment that shapes emotional burden and recovery after distressing events. These dimensions are not fully captured by alarm counts, response times, or nuisance reduction alone. To address this practical digital health gap, this qualitative interview study used digital nursing gaze and vicarious trauma as sensitizing lenses to explore how ICU nurses in a tertiary hospital in China experienced continuous monitoring and alarms in relation to attention, visibility, perceived accountability, emotional strain, and recovery after distressing events. The study aimed to generate design-relevant evidence for the human-centered design of monitoring displays and alarm prioritization and escalation logic and to inform future AI-supported monitoring systems that enhance patient safety while supporting clinicians’ work and recovery.

## Methods

### Study Design

This qualitative interview study explored ICU nurses’ experiences of continuous monitoring, frequent alarms, and vicarious trauma–related emotional strain in a digital critical care environment. We used a qualitative interpretive design with one-on-one semistructured interviews and analyzed the data using reflexive thematic analysis. The study is reported in accordance with the COREQ (Consolidated Criteria for Reporting Qualitative Research) [21] (Checklist 1).

### Setting

The study was conducted in the integrated ICU of a tertiary hospital in Hangzhou, Zhejiang Province, China. The unit is characterized by high-acuity care supported by continuous monitoring and frequent alarms from multiparameter bedside monitors, infusion pumps, ventilators, and other life-support devices. To minimize interruptions and ensure privacy, interviews were conducted in a private meeting room within the ICU department.

### Participants and Sampling

We used purposive maximum-variation sampling to recruit ICU nurses with varied roles and monitoring responsibilities. Sampling aimed to capture diversity in years of ICU experience, professional title (junior, intermediate, or senior), clinical role (eg, staff nurse, team leader, and specialized roles such as extracorporeal membrane oxygenation team member), sex, and exposure to high-intensity rescue situations or end-of-life care. Participants were eligible if they were registered nurses currently working in an adult ICU, routinely exposed to physiological monitoring and alarms, and able to complete a 40‐ to 60-minute interview in Chinese. Nurses on long-term leave during the study period or unable to complete an interview were excluded. Recruitment and preliminary analytic review proceeded iteratively. After the initial interviews, the research team reviewed whether the sample captured sufficient variation in ICU experience, professional title, clinical role, and exposure to high-impact monitoring or end-of-life events. Additional participants were recruited to strengthen variation and examine emerging patterns. A total of 15 ICU nurses participated. Recruitment ended when the dataset was judged sufficient to support coherent theme development in reflexive thematic analysis, with reference to the principle of information power [22,23].

### Recruitment

Recruitment occurred between November and December 2025. Potential participants were approached through direct invitation based on the purposive maximum-variation sampling strategy described above. To reduce perceived pressure to participate, the invitation emphasized that participation was voluntary, that declining would have no consequences for their employment, evaluations, or workplace relationships, and that participants could withdraw at any time without explanation. To support spontaneity and reduce rehearsed responses, only the general study aims were provided before the interview, with full topic prompts introduced during the conversation. Two nurses declined participation due to scheduling constraints; no participants withdrew after consenting.

### Interview Guide Development and Data Collection

The interview guide was developed based on the literature on alarm-intensive work, alarm management, nursing surveillance, vicarious trauma, and occupational distress in critical care settings; the study aim; and iterative team discussion to ensure clarity, sensitivity, and clinical relevance [24]. Interviews were conducted by 2 researchers: YW (female, MSc in Nursing, clinical research nurse) and XG (female, BSc in Nursing, clinical nurse). Both interviewers had qualitative research experience and no supervisory, managerial, or evaluative relationship with the participants.

Interviews were conducted in Chinese using a semistructured format and lasted 40 to 60 minutes. No nonparticipants were present during the interviews. The interview guide elicited accounts across 4 domains: everyday monitoring routines and alarm exposure; responses to alarms, patient deterioration, and death; perceived pressure related to visible physiological data and residual experiences after shifts, such as intrusive recall or lingering alarm sounds; and coping strategies and suggestions for improving alarm and monitoring systems. Interviews were audio-recorded with consent and transcribed verbatim. Transcripts were not returned to participants for comment or correction. Identifying information was removed during transcription. Transcripts were checked by the research team for completeness and accuracy before analysis, and each transcript was assigned a participant ID (P1-P15). The finalized interview guide is provided in Multimedia Appendix 1.

### Data Analysis

Data were analyzed using Braun and Clarke reflexive thematic analysis [23,25], with NVivo 15 used to support data organization and retrieval. Analysis proceeded iteratively alongside data collection. The first author led transcript familiarization, coding, and theme development, supported by analytic memos, reflexive notes, and field notes recorded after interviews. Initial coding was conducted inductively while remaining attentive to the study aim and participants’ accounts of monitoring routines, alarm-related events, emotional responses, perceived accountability, and recovery after distressing events. The theoretical concepts introduced in the Introduction informed the interview guide and later interpretation, but they were not used as predefined codes or themes; coding remained primarily inductive and grounded in participants’ accounts. Team meetings were used to discuss emerging codes, candidate themes, divergent accounts, and alternative interpretations. Coding differences were treated as prompts for reflexive discussion rather than as a basis for intercoder reliability, consistent with reflexive thematic analysis. Exemplar quotations were selected to illustrate recurrent patterns and meaningful variation across participants. Quotations were translated into English for reporting, with attention to preserving meaning, nuance, and professional tone.

### Trustworthiness and Reflexivity

Trustworthiness was supported through reflexive documentation, field notes, peer debriefing, and an audit trail of recruitment, coding, theme development, and analytic decisions. Team discussions were used to examine emerging interpretations, divergent accounts, and theme boundaries. Thick description of the ICU setting, participant characteristics, monitoring context, and illustrative quotations supported transferability. Participants were not asked to provide feedback on the final themes or findings.

Reflexivity focused on how the researchers’ nursing and clinical research backgrounds may have shaped data generation and interpretation. These backgrounds helped build rapport and supported understanding of ICU monitoring routines but may also have sensitized the team to alarm burden, accountability, and distress after deterioration or death. To manage these influences, interviewers used open-ended prompts, and the team used memos and discussions to examine assumptions about monitoring, responsibility, emotional endurance, and good nursing.

### Ethical Considerations

Ethical approval was obtained from The First Affiliated Hospital of Zhejiang Chinese Medical University (2025-KLS-184‐02). All participants provided informed consent. Participation was voluntary, and participants could pause or terminate the interview at any time. No financial compensation or other incentives were provided. Given the emotionally sensitive nature of vicarious trauma narratives, interviews were conducted with attention to distress cues, and breaks were offered as needed. All data were deidentified and stored securely.

## Results

### Overview of the Findings

Participants described working in ICU environments organized around continuous physiological monitoring, central displays, frequent alarms, and work-related digital communication. Reflexive thematic analysis generated 4 themes and 12 subthemes: (1) digital gaze, (2) digitally mediated vicarious trauma, (3) after-shift residue, and (4) recovery strategies and system-level needs. Demographic characteristics of the participants are presented in Table 1.

**Table 1.** Demographic characteristics of the participants (N=15).

Characteristics		Participants, n (%)
Age (years)		
18‐25		1 (6.7)
26‐30		5 (33.3)
31‐35		3 (20)
≥36		6 (40)
Sex		
Male		3 (20)
Female		12 (80)
Marital status		
Unmarried		6 (40)
Married		9 (60)
Education level		
Associate degree		4 (26.7)
Bachelor’s degree		10 (66.7)
Master’s degree		1 (6.7)
Critical care experience (years)		
<5		3 (20)
5‐10		8 (53.3)
>10		4 (26.7)
Professional title		
Junior		10 (66.7)
Intermediate		5 (33.3)
Role		
Staff nurse		11 (73.3)
Team leader/deputy team leader		3 (20)
ECMO^a^ team member		1 (6.7)

a ECMO: extracorporeal membrane oxygenation.

### Theme 1: Digital Gaze—Attention, Visibility, and Work Rhythms Under Continuous Monitoring

#### Overview

This theme describes how continuous monitoring and alarms shaped where nurses looked, how quickly they responded, and how responsible they felt when physiological parameters changed. Participants described monitors and alarms not as passive background devices but as technologies that directed attention, made unstable data visible to others, and repeatedly interrupted bedside work.

#### Subtheme 1.1: Constant Watching and Hypervigilance

Participants described continuous monitoring as creating a state of constant watching, especially when vital signs were unstable or patients could deteriorate suddenly. Nurses felt they had to keep part of their attention on the monitor even while performing other care tasks because missing a change could have serious consequences.

*I was doing nursing care while staring at the monitor...the vitals fluctuated so much that if you didn’t watch closely, you feared missing a critical change*.

[P11]

This vigilance was therefore embedded in everyday monitoring routines rather than experienced as a separate task.

#### Subtheme 1.2: Surveillance Pressure and Perceived Accountability

Participants described pressure arising from the visibility of physiological data. When parameters were unstable, monitor screens became shared points of attention for nurses and physicians. In these moments, nurses felt that visible numbers could make their bedside responses open to judgment, especially when the patient’s condition was difficult to stabilize.

*Doctors stood next to me staring at the screen, urging me to adjust vasopressors…I have to admit, I’m even more afraid of being blamed*.

[P5]

For some nurses, the monitor made both patient instability and nursing performance visible:

*I watched the monitor nonstop...I feared doctors would think my vital-sign management was poor. At the bedside, you’re the one who gets seen first*.

[P11]

A senior participant summarized this tension as a mismatch between responsibility and control:

*Responsibility is huge, but power is limited—you carry outcome pressure without outcome power*.

[P15]

#### Subtheme 1.3: Alarm-Driven Interruptions and Fragmented Work Rhythms

Frequent alarms repeatedly interrupted nursing tasks and forced nurses to shift their attention among ongoing care, monitor screens, infusion pumps, and alarm responses. Participants described this as the fragmentation of work rhythm rather than simply increased noise.

*You keep getting interrupted—start a procedure, an alarm goes off; write two lines, then the pump alarms again*.

[P5]

A senior nurse emphasized that repeated alarms could prevent the completion of a coherent workflow:

*It’s not one alarm—handled it and it goes off again. Your attention gets chopped up, and you can’t complete a full workflow*.

[P15]

### Theme 2: Digitally Mediated Vicarious Trauma—Emotional Strain During Monitored Deterioration

#### Overview

This theme describes nurses’ emotional strain when patient deterioration and death were encountered through alarms, waveforms, and visible physiological data. Participants emphasized that monitoring technologies could make deterioration more immediate and harder to distance themselves from, especially when close observation and advanced equipment could not change the outcome.

#### Subtheme 2.1: Monitored Deterioration and Screen-Visible Death

Participants described deterioration and death as processes often witnessed through changing waveforms, escalating alarms, numerical decline, and eventual silence. These monitor-mediated signs made decline emotionally striking because nurses could see and hear the process unfolding while also recognizing that it might be irreversible.

One participant described how weakening waveforms and repeated alarms made deterioration visibly and acoustically present before death was formally recognized:

*The waveform got weaker and alarms became more frequent... deep down you know it’s often irreversible*.

[P13]

For other nurses, emotional strain was intensified by the contrast between a patient’s earlier interaction and sudden deterioration:

*He was talking when he arrived, and then suddenly we were resuscitating him...that contrast hit me hard*.

[P9]

#### Subtheme 2.2: Technological Limits and Powerlessness

Participants described powerlessness when close monitoring and advanced equipment could not prevent deterioration or death, even when technical care was appropriate. Technology could provide detailed information and support life-sustaining treatment, but it did not always translate into recovery.

*We could keep the equipment optimal, but we couldn’t ‘keep the person’...the machine ran perfectly, but the patient didn’t come back*.

[P8]

A senior nurse summarized the same tension more directly:

*Monitoring matters, but monitoring doesn’t mean the outcome will get better*.

[P15]

#### Subtheme 2.3: Maintaining Parameters Under Limited Control

Participants described strain when they had to respond to unstable parameters while having limited control over treatment decisions or patient outcomes. This was especially difficult when nurses were positioned between patient safety, family decisions, and treatment limits. In such situations, maintaining acceptable numbers could feel like a continuous effort to contain instability without being able to resolve the underlying problem.

*You’re caught in the middle: you can’t decide for the family, but you’re responsible for patient safety. That push-and-pull is exhausting*.

[P10]

In some accounts, physiological numbers also became tied to family anxiety:

*At that moment, I wasn’t only caring for the patient—I was holding the numbers for the family’s fear*.

[P3]

### Theme 3: After-Shift Residue—Lingering Alarm Sounds, Monitor Images, and Personal Resonance

#### Overview

This theme describes how monitoring-related stress sometimes continued after nurses left the ICU. Participants described lingering alarm sounds, monitor images, bodily alertness, work-related messages, and personal life experiences that shaped how they remembered and recovered from distressing events.

#### Subtheme 3.1: Lingering Alarm Sounds and Monitor Images

Participants reported lingering sensory traces after shifts, including alarm sounds, monitor images, waveforms, and physiological alertness. These experiences were often involuntary, repetitive, and difficult to switch off after leaving the unit.

*At home, I was exhausted, but it still felt like alarms were ringing in my ears...like my brain was auto-replaying it*.

[P1]

Participants also described specific monitor images that remained after high-impact events:

*After a failed resuscitation, that waveform and alarm sound stay with you for a long time*.

[P15]

#### Subtheme 3.2: Personal Resonance and Heightened Sensitivity

Some participants described stronger emotional reactions when ICU events resonated with their own life experiences, such as reproductive loss, family hospitalization, or caregiving responsibilities. These accounts suggest that emotional responses to monitoring-related events were shaped not only by professional exposure but also by nurses’ personal histories and current life situations.

*I’ve had multiple pregnancies that ended in miscarriage...it makes me more sensitive to life being cut off so suddenly*.

[P7]

Another participant described how hearing monitor sounds during a parent’s hospitalization changed the meaning of those sounds:

*When my parents were hospitalized, I heard the same monitor sounds again...you’re not only a nurse, you’re also a family member*.

[P2]

#### Subtheme 3.3: Work-Related Digital Spillover After Shifts

Work-related messages and notifications kept some nurses connected to ICU events after their shifts, making psychological detachment incomplete. Unlike alarm sounds or monitor images that remained in memory, this form of after-shift stress was triggered by ongoing digital communication. Messages in work groups could bring nurses’ attention back to unstable patients, resuscitations, or urgent unit events even when they were physically away from the ICU.

One participant described the phone as immediately pulling attention back to ICU work:

*My phone is like a string pulling me back to the ICU...it vibrates and my attention snaps back immediately*.

[P1]

Another participant explained that even brief messages about an ongoing resuscitation could restart worry and attention:

*Once someone posts ‘bed X is being resuscitated,’ I immediately think: who is it, what happened? I can’t disconnect completely*.

[P2]

### Theme 4: Recovery Strategies and System-Level Needs

#### Overview

This theme describes how nurses sought to recover from monitoring-related stress through professional boundaries, everyday coping strategies, and meaning rebuilding. At the same time, participants emphasized that recovery should not depend solely on individual coping. They also identified system-level changes needed in alarm design, alarm prioritization, and postevent working conditions.

#### Subtheme 4.1: Professional Boundaries and Self-Protection

Participants described professional boundaries, careful procedures, and documentation as ways to protect themselves while continuing to work in the ICU. These strategies helped nurses reduce rumination and maintain a sense of professional control in a work environment where outcomes were often uncertain.

*At work, I do everything to be beyond reproach; after work, I cut it off. That’s self-protection*.

[P11]

Participants did not describe boundaries as emotional numbness. They also emphasized the need to remain clinically responsive to meaningful patterns:

*You can’t be terrified by every alarm, but you have to stay sensitive to trends and dangerous combinations*.

[P15]

#### Subtheme 4.2: Multilayered Pathways to Resilience and Meaning Rebuilding

Nurses described everyday recovery strategies such as gaming, traveling, reading, and spending time with others. These activities helped them shift their attention away from distressing events, restore emotional energy, and reframe what they could still control in their professional roles.

*Gaming helps me switch off. It pulls my attention somewhere else instead of replaying the resuscitation*.

[P9]

For some participants, reading provided a way to make sense of uncontrollable outcomes while maintaining professional responsibility:

*Reading helps me find an explanation—many things can’t be controlled, but I can still be accountable in my role*.

[P12]

#### Subtheme 4.3: System-Level Improvement Needs

Participants emphasized that recovery should not depend solely on individual coping. They suggested improving alarm systems through clearer alarm levels, fewer nonactionable alarms, gentler alarm sounds, trend-based displays, and buffering modes after high-impact events.

One participant described the need for a more humane alarm system that could distinguish urgency and reduce unnecessary alarm pressure after resuscitation:

*I want the system to be more human: clearer alarm levels, softer sounds, and a buffer mode after resuscitation so low-value alarms don’t keep crushing you*.

[P2]

Another participant connected smarter alarm logic with trend judgment and reduced low-value interruptions:

*Alarms should be smarter—not everything should sound. The system should judge trends and reduce meaningless alarms. Different alarm levels should be clearer, and after resuscitation, there could be a buffer mode that shows key indicators quietly so we are not bombarded by many minor alarms*.

[P1]

Participants also emphasized that alarms should support clinical interpretation rather than simply produce more signals:

*Reduce false alarms and make alarms more valuable…show trends so decisions aren’t based on a single moment*.

[P14]

## Discussion

### Principal Findings

Continuous monitoring functioned not only as a safety technology but also as an infrastructure that organized attention, visibility, responsibility, and the sensory experience of deterioration. The findings suggest that alarm-intensive care should be understood as a sociotechnical design issue in which displays, alarm behavior, communication platforms, and emerging AI-supported tools shape both patient safety work and clinicians’ recovery. The discussion therefore focuses on how monitoring systems should represent clinical transitions, govern alarms, protect recovery time, and avoid creating new forms of surveillance.

### Digital Monitoring Beyond Alarm Fatigue: Attention, Visibility, and Technology-Mediated Vicarious Trauma

Our findings suggest that continuous monitoring functioned not simply as a safety technology or a source of alarm fatigue but as an infrastructure that organized attention and work rhythms. Nurses described maintaining a peripheral watch while performing bedside tasks, while frequent alarms fragmented care, documentation, and decision-making. Consistent with alarm-fatigue research on mental workload and alarm relevance [26-28], the monitor-alarm system redistributed attention among the bedside, screens, infusion devices, and emerging alarms.

Monitoring also made patient instability and nursing work simultaneously visible. In our data, when deterioration was difficult to reverse, visible values could heighten nurses’ sense that their bedside responses were open to scrutiny despite their limited control over treatment decisions or patient outcomes. Although shared monitoring displays can support situation awareness and interdisciplinary coordination [29,30], our findings suggest that the same visibility may also create accountability pressure. Digital gaze, therefore, helps explain how display design, alarm behavior, team hierarchies, and critical illness jointly shape visibility and perceived accountability [9-11].

Beyond these everyday work effects, monitoring also mediated nurses’ encounters with deterioration and death. In this study, monitor-generated sensory traces formed part of how deterioration was anticipated and remembered. This extends evidence that ICU professionals exposed to suffering, deterioration, and death may experience trauma-related symptoms or secondary traumatic stress [16,31] and that intrusive memories may persist after work-related traumatic events [32]. Rather than independently causing vicarious trauma, monitoring technologies may shape the sensory and interpretive conditions under which high-stakes events are witnessed.

### Designing for High-Impact Clinical Transitions: Deterioration, Resuscitation, and Death

Participants’ accounts suggest that monitoring systems are not neutral during high-impact clinical transitions. In our data, weakening waveforms, escalating alarms, numerical decline, and subsequent silence marked the movement from active deterioration or resuscitation toward a postevent or end-of-life state. This raises a distinct design question: how should monitoring systems behave when clinical status and care goals are changing? Human factors research on patient-monitor interfaces emphasizes designs that support real-time interpretation of evolving physiological information and safe, efficient monitor use [12,33]. Our findings extend this concern by suggesting that interfaces should make changes in clinical and care status legible rather than simply presenting successive abnormalities.

This design challenge is particularly salient at the end of life. Participants’ suggestion of a postresuscitation buffer mode reflects a perceived mismatch between routine alarm behavior and the clinical context after resuscitation termination or death. End-of-life alarm research supports this concern: arrhythmia alarms may remain audible after comfort-care initiation [34], and ongoing telemetry alarms can interfere with end-of-life conversations and family interaction [35].

The proposed buffer mode should therefore be regarded as a data-grounded design hypothesis rather than a ready-to-deploy solution. Future participatory work could examine a clinician-initiated, workflow-governed postevent mode following resuscitation termination, confirmed death, or other end-of-life transitions, with users, workflows, and real-world data informing its development [2]. Because withdrawal of life-sustaining treatment and end-of-life transitions require protocolized clinical processes, any such mode should follow clinical confirmation rather than automatically inferring death [36]. It should preserve safety-critical alarm escalation while reducing repetitive low-priority alerts and making relevant device status and immediate next tasks more visible [5].

### From Alarm Governance to Nonsurveillant Digital Support

At the system level, the implication is not simply to reduce alarms. Participants described competing alerts, repeated interruptions, and uncertainty about alarm relevance. Studies of patient-specific alarm customization and intelligent alarm management likewise indicate that alarm burden can be reduced when configurations are adapted to the clinical context while preserving safety [37,38]. Our findings add that effective alarm governance should be aligned with clinical workflows: alarm settings should be patient- and context-sensitive, alarm burden may need periodic review, and responsibility for reviewing, adjusting, and responding to alarms should be explicit across the care team.

Digital support should also respect the temporal boundary of work. Participants described work-group messages and phone notifications as drawing attention back to unstable patients and resuscitations after shifts. This aligns with recovery research identifying psychological detachment as central to recovery from job stress and with evidence linking work-related smartphone use during off-job hours to greater work-life conflict [39,40]. Communication platforms should therefore use role-sensitive escalation pathways and clear expectations for after-hours messaging so that urgent information reaches the appropriate on-duty clinician without routinely extending nonurgent monitoring into recovery time.

Finally, AI-enabled alarm support or wellbeing tools should be designed as assistance rather than another layer of observation. Although participants did not discuss AI directly, their accounts of visible data, scrutiny, and accountability indicate that systems inferring workload, emotional state, or performance may be experienced as surveillance, particularly when their outputs are accessible to managers or linked to appraisal. This concern is consistent with workplace surveillance research showing that perceived surveillance is associated with psychological distress and lower job satisfaction through increased job pressure, reduced autonomy, and privacy concerns [41]. Consistent with health AI ethics and governance guidance, such tools should clearly disclose their purpose, data sources, access arrangements, and intended uses, and should preserve clinicians’ ability to question or override outputs [42]. In this context, wellbeing data should be kept separate from performance management, and nurses should be involved in co-design and evaluation [43]. Evaluation should assess perceived surveillance and staff experience alongside technical performance and patient safety.

### Limitations

Several limitations should be noted. This study was conducted in a single tertiary ICU in China, which may limit transferability to other organizational, technological, and cultural contexts. Participants were recruited through direct invitation, which may have introduced self-selection bias, as nurses who agreed to participate may have been more willing to discuss emotionally sensitive experiences or more reflective about alarm-related work. As with interview-based research, accounts may be shaped by recall bias, social desirability, or recent emotionally salient events, although confidentiality and the absence of supervisory or evaluative relationships likely supported openness. Finally, quotations were translated from Chinese into English, and some linguistic or cultural nuances may have been altered despite efforts to preserve their meaning.

### Conclusion

This qualitative interview study suggests that continuous monitoring and frequent alarms shape ICU nurses’ attention, perceived accountability, emotional strain during monitored deterioration, and recovery after shifts. Alarm-intensive care should therefore be approached as a digital health and sociotechnical design issue as well as a patient safety concern. Alarm governance, display design, work-related communication, and future AI-supported monitoring should be co-designed and evaluated for patient safety, alarm reduction, clinicians’ attention and recovery, and perceived surveillance.

## Supplementary material

10.2196/91807 Multimedia Appendix 1 Interview guide.

10.2196/91807 Checklist 1 COREQ checklist.

## Abbreviations

COREQ: Consolidated Criteria for Reporting Qualitative Research
ICU: intensive care unit
