# Supplementary material for: Digital Gaze and Vicarious Trauma Among Intensive Care Unit Nurses in Alarm-Monitoring Ecologies: Qualitative Interview Study
Source: J Med Internet Res. 2026 Aug 4;28:e91807. doi: 10.2196/91807 (PMC13436847; doi:10.2196/91807)
Supplement: Supplementary file 1 [file jmir-v28-e91807-s001.docx]

Interview Guide

Q1: Please briefly introduce your years of ICU experience, your current role, and your main duties

Q2: Please describe your daily work with monitoring and alarm systems. How does the sensory environment differ across shifts and between beds?

Q3: Please recall one event involving alarms, monitoring, or sudden parameter changes that left a strong impression on you (eg, you still think about it after work).

Q4: Do you see the monitor/alarm system as a kind of "digital gaze"? How does it compel your attention or make it hard to look away? What does that mean to you?

Q5: Which types of alarms are most likely to trigger tension, helplessness, or fear? When alarms are dense, how do your tasks and teamwork change?

Q6: In your view, what factors amplify or buffer alarm-related stress?

Q7: Has the continuous monitor-alarm context ever led you to feel numb, avoidant, or hypervigilant?

Q8: After work, do alarm sounds or parameter changes ever "follow" you home (eg, intrusive memories, imagined sounds)?

Q9: If future monitoring systems could be redesigned, what emotional-protection features would you most want to add?

Q10: Would you be willing to use digital support tools? What features should an ideal tool/service have? What risks worry you the most (eg, privacy, stigma, being evaluated, increased burden)?

Q11: Is there anything important that we did not ask but you think matters?
